# Using Electronic Medical Records to Identify Enhanced Recovery After Surgery Cases

**DOI:** 10.5334/egems.304

**Published:** 2019-07-26

**Authors:** Nikki L. B. Freeman, Katharine L. McGinigle, Peter J. Leese

**Affiliations:** 1 University of North Carolina at Chapel Hill, US; 2 University of North Carolina School of Medicine, US; 3 University of North Carolina Center for Health Innovation, US

**Keywords:** Electronic health records, Integrated delivery of health care, interdisciplinary communication, perioperative care, enhanced recovery after surgery

## Abstract

**Context:**

Enhanced recovery after surgery (ERAS) aims to improve surgical outcomes by integrating evidence-based practices across preoperative, intraoperative, and postoperative care. Data in electronic medical records (EMRs) provide insight on how ERAS is implemented and its impact on surgical outcomes. Because ERAS is a multimodal pathway provided by multiple physicians and health care providers over time, identifying ERAS cases in EMRs is not a trivial task. To better understand how EMRs can be used to study ERAS, we describe our experience with using current methodologies and the development and rationale of a new method for retrospectively identifying ERAS cases in EMRs.

**Case Description:**

Using EMR data from surgical departments at the University of North Carolina at Chapel Hill, we first identified ERAS cases using a protocol-based method, using basic information including the date of ERAS implementation, surgical procedure and date, and primary surgeon. We further examined two operational flags in the EMRs, a nursing order and a case request for OR order. Wide variation between the methods compelled us to consult with ERAS surgical staff and explore the EMRs to develop a more refined method for identifying ERAS cases.

**Method:**

We developed a two-step method, with the first step based on the protocol definition and the second step based on an ERAS-specific medication definition. To test our method, we randomly sampled 150 general, gynecological, and urologic surgeries performed between January 1, 2016 and March 30, 2017. Surgical cases were classified as ERAS or not using the protocol definition, nursing order, case request for OR order, and our two-step method. To assess the accuracy of each method, two independent reviewers assessed the charts to determine whether cases were ERAS.

**Findings:**

Of the 150 charts reviewed, 74 were ERAS cases. The protocol only method and nursing order flag performed similarly, correctly identifying 74 percent and 73 percent of true ERAS cases, respectively. The case request for OR order flag performed less well, correctly identifying only 44 percent of the true ERAS cases. Our two-step method performed well, correctly identifying 98 percent of true ERAS cases.

**Conclusion:**

ERAS pathways are complex, making study of them from EMRs difficult. Current strategies for doing so are relatively easy to implement, but unreliable. We have developed a reproducible and observable ERAS computational phenotype that identifies ERAS cases reliably. This is a step forward in using the richness of EMR data to study ERAS implementation, efficacy, and how they can contribute to surgical care improvement.

## Context

Enhanced recovery after surgery (ERAS) is an increasingly used, comprehensive clinical pathway that standardizes and coordinates evidence-based best practices across specialties to achieve early recovery for patients undergoing major surgery [1,2]. ERAS pathways have been implemented in cardiac, head and neck, colorectal, hepatobiliary, gastric, gynecologic, and urologic surgical services with the aim to ensure the delivery of evidence-based treatments by a diverse group of physicians and other health care providers [1]. The rationale of ERAS is that surgical outcomes, particularly length of stay and surgical complications, depend not only on the surgical procedure itself, but on the entire surgical process including preoperative, intraoperative, and postoperative care. While each pathway is specific to its respective surgical specialty, there are shared, fundamental concepts between all pathways. These shared concepts include preoperative patient education and expectation-setting, preoperative medical and nutritional optimization, pre-emptive multi-modal analgesia, an intraoperative goal-based fluid strategy, postoperative opioid minimization, pre-emptive anti-emetic and bowel regimen, early drain and line removal, early mobilization, and early resumption of regular diet [3].

The use of electronic medical records (EMRs) is widespread [4], and can provide an opportunity to understand how ERAS protocols are implemented and their potential impact on surgical outcomes. However, accurately identifying cohorts from observational datasets or operational data systems like EMRs can be more challenging than prospective data collection. Because ERAS is a medical, metabolic, physical, and emotional care pathway provided by a coordinated group physicians and health care providers over time, identifying ERAS cohorts has not been easily distilled into a discrete flag or check-marked box in a health record. As a result, identifying ERAS patients retrospectively in an EMR has proven challenging and unreliable using EMR proxies for ERAS compliance, such as orders and procedure scheduling documentation. At present, most retrospective ERAS studies are historical cohort studies, identifying patients as having received ERAS based on the timing of their surgical procedure and the timing of ERAS implementation [5,6,7,8,9]. That is, all patients undergoing surgery after the adoption of ERAS by a particular surgeon or surgical team for a particular operation are classified as ERAS patients regardless of whether ERAS procedures were implemented fully, partially, or not at all. Lemini et al. used a method to assess ERAS compliance after pathway implementation, but did not use their compliance score to identify ERAS patients [10]. Additionally, some providers maintain manually curated registries to identify ERAS cohorts, track pathway compliance, and follow-up on short and long-term outcomes. However, a registry approach requires resources to build and maintain the registry system, additional resources to enter and curate the data, and this manual process does not scale effectively for large or established ERAS programs with hundreds or thousands of cases to abstract. As a result, the use of registries has not been a quick or easy method for assembling ERAS datasets for clinical care, operations, or research.

Understanding the details of ERAS pathway utilization including how and when they are used, for which types of patients, and by which surgeons can provide important information in how to improve the quality of care, safety, and outcomes of surgical patients. Due to lack of an existing gold-standard identification method for retrospective ERAS identification, the obstacles associated with registry collection, and the over-reliance on convenience-based methods of identification, we sought to develop and observational method which could be employed as an ERAS computable phenotype. In the following, we detail our journey in developing an EMR-observable ERAS phenotype. We compare existing ERAS identification methods to our proposed phenotype on a sample of surgical patients from a large academic medical center.

## Case Description

### Setting

The University of North Carolina (UNC) Medical Center is a public academic medical center in Chapel Hill, North Carolina. With more than 900 beds, the UNC Medical Center provides patient care, engages in medical research, and educates health care professionals in partnership with the UNC Schools of Medicine, Nursing, Pharmacy, and Public Health. The surgical departments at UNC perform over 35,000 procedures a year, providing clinical services to patients in North Carolina and throughout the Southeastern United States. The UNC Medical Center internally developed, implemented, and maintained a web-based EMR in 1991. This system was further refined and updated until it was purchased by Siemens in 2009. Since 2014, the entire, multi-hospital UNC Healthcare system, including the Medical Center, has used an Epic EMR system.

### Journey

Our interest in identifying ERAS patients from the EMR began with a desire to evaluate, inform, and improve the ERAS program at UNC. Early in our efforts, we found that creating a credible, reproducible ERAS cohort from EMR data was both non-trivial and essential to the scientific rigor we hoped to bring to our task. Much of this difficulty stems from the fact that ERAS is implemented in EMR in an organic, multi-faceted manner requiring both deep clinical knowledge of program and surgical details and also deep technical knowledge of EMR function, data modeling, and data retrieval.

Two key researchers, Mr. Leese and Dr. McGinigle, formed the core team for this effort and provided the necessary knowledge and skills. Mr. Leese is a data scientist with extensive experience in accessing and analyzing EMR data for clinical epidemiology. Dr. McGinigle is a faculty surgeon and the surgical director of the ERAS program at UNC. Together, Dr. McGinigle’s bird’s-eye view of the ERAS program and Mr. Leese’s ability to operationalize and analyze various ERAS patient identification methods contributed to our progress in our effort. Knowledge between the two was integrated iteratively through in-person and electronic communications at each step to ensure that technical and clinical objectives were in concordance.

When we began considering various approaches for retrospectively identifying ERAS patients from EMR, we first used the previously described protocol-based method to identify possible ERAS cases from all surgical cases performed. To further refine and characterize these possible cases we subsequently investigated two operational flags in the EMR: a nursing order and a surgical case request form button. The specific nursing order identified is used at UNC to indicate patient intent to follow an ERAS protocol for a scheduled surgery, and the case request button is a binary yes/no checkbox added to the operating room case request form in the EMR to indicate the intention for a scheduled surgery to follow an ERAS protocol. To the best of our knowledge both the order and case request button were built sometime after the use of ERAS pathways started at UNC. We describe each of these in this section.

We initially codified the details of each surgery-specific ERAS protocol at UNC, and implemented these code-based rules in a SQL program to extract the primary surgeon, each Current Procedural Terminology (CPT) code, clinical service, and surgical date from the EMR. The extracted EMR elements from surgical cases were compared to the codified rules based on the elements from each ERAS protocol to determine if a given surgery was a possible ERAS case. This method identified a possible 4,871 ERAS operations over an approximately 4-year period (7/2014 to 9/2018). From our review it appears many published historical cohort studies examining ERAS outcomes and processes, identified simple cohorts in this manner.

We next examined and characterized the use of ERAS nursing orders in the EMR to further investigate the status of the possible ERAS cases identified. At the most granular transactional level (patient, encounter, order number), 9,420 orders assigning a case to ERAS were identified; however, when only the patient/encounter level was considered the number of orders decreased to 8,962. This indicates that for some portion of surgical cases duplicate or multiple orders have been entered, and cumulatively this same behavior occurred hundreds of times. Additionally, upon further review it became apparent that several thousand additional nursing orders for ERAS existed that could not be matched to one of our identified possible ERAS cases. The exact etiology of these issues is not fully known, but inappropriate use of the order is suspected. Further muddling the matching process, the timing of the ERAS order was often days or weeks before the actual surgical date, making it difficult to link to the intended surgical procedure using the EMR linkage keys at the order and encounter level. As a result, when we attempted to link specific instances of ERAS nursing orders to respective operations, only a fraction of matches where successfully made.

Next, we examined the use of case request forms (CRF) indicating an intent to follow an ERAS protocol for a surgery. Unlike orders, CRF forms are transacted at the same linkage level as surgical cases (case ID as opposed to encounter), facilitating the confident linkage between a CRF and an operation. At the OR case ID level 6,389 case request forms were identified indicating an intent to follow an ERAS protocol at the time of submitting the case request. This is several hundred additional ERAS cases beyond the identified potential cases. When examining the use of CRF forms in more detail, we identified that of the ERAS cases actually eligible by the protocol method (4,871) about half (2,418) had some type of CRF ERAS selection. Within those 2,418 the actual CRF values included 741 instances of a code indicating a value of ‘zzz’ and 1,677 instances of ‘yes’. It is unclear, but our belief is that the CRF value of ‘zzz’ was created initially to test the ERAS functionality of the CRF, and somehow this value was inadvertently used in the production EMR for some time period; however, at some point, it appears that the CRF ERAS value was switched to the intended value of ‘yes’. When examining the approximately 4,000 CRF forms that did not match the eligible ERAS cases identified using the protocol-based method, a variety of issues were identified. First, a portion of CRF forms indicating an intent to follow an ERAS pathway were for surgeries performed at eligible institutions, but for surgeons, procedures, and clinical services that do not have institutionally-recognized and approved ERAS protocols. It is possible these services have informal ERAS practices that have not been approved or reconciled with existing ERAS protocols, or that these were simple errors. Additionally, since the same EMR is available at all UNC Healthcare system hospitals in addition to the UNC Medical Center, we found that some ineligible uses of the ERAS indicator on the CRF form arose from usages by individual surgeons at outlying hospitals that do not yet have a formal ERAS program.

After assessing both case request forms and ERAS orders, we strongly believed that neither operational tool could be used to accurately identify ERAS surgeries from the EMR. Following this decision, we began to develop a new method that could lead to a usable ERAS computable phenotype for use in observational data. Ideally, a comprehensive phenotype could be identified that is inclusive of all elements of an ERAS pathway, covering pre-surgery preparation, perioperative and operative surgical adherence, and post-operative factors; however, some of these factors are not documented in EMR in a consistent, discrete way making their usefulness limited for an electronic algorithm. Following the consideration of multiple possible criteria for building such an algorithm, we settled on using administration of immediate pre-operative, multi-modal analgesia in the medication administration record (MAR) as the most definitive method of determining whether an eligible operation actually adhered to an ERAS protocol. We decided on this criteria since these medications are universal in all ERAS pathways across surgical specialties and are ordered as part of the pre-operative order set, indicating a priori thought and intent to include the patient in an ERAS clinical pathway.

While the specific details of ERAS implementation and requisite EMR data may differ at other institutions, we believe the algorithmic process described in our journey is generalizable to other institutions, regardless of EMR system or ERAS implementation. At the most fundamental level, the concept of combining surgical data with ERAS-specific medication compliance data should be a near-universal model for identifying ERAS patients, and it is the fundamental nature of this criterion that appealed to us when considering various approaches.

## Method

Following review of the accepted ERAS protocols, discussion with clinical ERAS staff, and exploration of EMR data, we found that identifying patients with near-concurrent administration of at least two of three ERAS medications (acetaminophen, pregabalin, and celecoxib) prior to the start of an imminent surgery identified ERAS patients well. Specifically, we codified these criteria as receiving at least two of the three ERAS multi-modal analgesics within 5 minutes of each other and within 4 hours of surgery. Although we could have chosen any number of ERAS components (e.g. early ambulation) as markers of ERAS compliance, our rationale for selecting the course of medication in the immediate pre-operative period comes from a clinical standpoint and the belief that intentionally ordering the pre-operative medications as part of the pre-operative order in the pre-operative clinic where surgical risks, benefits, and expectations are reviewed is the most reliable way to show that physicians intend for their patients to follow an ERAS protocol. This approach, however, also identified many non-ERAS patients receiving a similar course of medication prior to surgery. We further refined our method by identifying ERAS patients as those that met our medication definition and also met the existing protocol-based definition. This two-step method enabled us to identify ERAS patients while eliminating patients that were either non-compliant on medications or failed to meet surgical criteria of one of our defined ERAS protocols.

To test the characteristics of our two-step method, we randomly sampled 150 general, gynecologic, and urologic surgeries performed at UNC Medical Center between January 1, 2016, and March 30, 2017. Cases were classified as having received ERAS using the protocol-based method, nursing order, the case request button, and using our two-step method (medication definition + protocol). To assess the accuracy of each method, two independent reviewers assessed the charts to determine whether cases were ERAS (surgeries eligible for ERAS and received the ERAS protocol) or not. Disagreements were reconciled through consensus.

## Findings

One-hundred and fifty charts were assessed, and 74 ERAS cases and 76 non-ERAS cases were identified. Classification percentages of the four approaches are summarized in Table 1. Using the protocol only method identified all 74 ERAS cases, however it also identified 26 non-ERAS cases as ERAS cases (34 percent misclassification of non-ERAS cases). This means that of the cases identified as ERAS by the protocol method, only 75 percent were consistent with ERAS protocols. This indicates that this criterion is too broad, and is consistent with our belief that this method should be refined with other criteria, such as the medication definition, to determine actual ERAS compliance.

**Table 1:** Classification of surgical cases as ERAS or non-ERAS by the four methods.

	Chart review ERAS cases (n = 74)		Chart review non-ERAS cases (n = 76)		Percent of cases identified as ERAS that were ERAS cases

	Identified as ERAS (% of chart review ERAS cases)	Identified as non-ERAS (% of chart review ERAS cases)	Identified as non-ERAS (% of chart review non-cases)	Identified as ERAS (% of chart review non-cases)	

Protocol only	74 (100%)	0	50 (66%)	26	74%
Nursing order	53 (72%)	21	56 (74%)	20	73%
Case request for OR order	33 (45%)	41	34 (45%)	42	44%
Medication definition + protocol	49 (66%)	25	75 (99%)	1	98%

Using the nursing order to identify ERAS cases had a 28 percent misclassification rate among ERAS cases identified by chart review and a 26 percent misclassification rate among non-ERAS cases identified by chart review. The other operational flag, the case request for OR order, had an even worse misclassification rate. It misclassified more than half of both ERAS cases and non-ERAS cases. Given the high rates of misclassification by these flags, we believe that neither are reliable indicators of ERAS pathway use or compliance.

Our two-step method using the medication criterion in addition to the protocol definition identified 49 of the true ERAS cases. Twenty-five cases identified as ERAS by chart review were misclassified as non-ERAS cases by the two-step method. Upon further review of these charts, we found clinical factors explaining the discrepancy, including patient allergies to the medication, patient refusal to take the medications, and physicians failing to order the medications despite ordering the other ERAS pathway compliant orders. More importantly, our method identified only 1 of the non-ERAS cases as an ERAS case. This means that of those identified as ERAS by the two-step method, 98 percent of them were actually ERAS cases as identified by chart review.

## Conclusion

ERAS pathways are complex and integrated protocols that span time and personnel, and this complexity makes studying them from the EMR particularly challenging. In our experience, operational flags such as the presence of a nursing order indicating the intention to follow an ERAS pathway or ERAS indicated in a case request for the OR are unreliable, prone to human error, and can be difficult to match to the intended surgical procedure in EMRs alone. Despite being a popular choice in identifying ERAS cases in EMR, protocol-based methods tend to have many false positives, identifying non-ERAS cases incorrectly as ERAS cases. To address these shortcomings, we developed a two-fold criterion for identifying ERAS cases in EMR that applies the protocol-based approach with a medication definition. We believe that the timing and setting of when the immediate pre-operative medication order is given is the most reliable way to show a physician’s intention for a patient to follow an ERAS protocol, and that the pre-operative medication profile is an indicator that can be reliably extracted from EMR data. This is unlike other parts of the clinical pathway, such as early ambulation, which are often applied to all patients, may not be intentional, and therefore less reliable for identifying ERAS patients. By using a protocol and medication definition together, 98 percent of the patients identified as ERAS patients actually received ERAS. Our method is not without limitations. In our sample some ERAS cases were erroneously classified as non-ERAS. These occurred when records failed to meet the medication criterion due to patients refusing medication, patients had medication allergies, and physicians forgot the medication orders despite completing all of the other orders consistent with ERAS. That is, there is some cost for our major improvement on the positive predictive value of case identification when compared to other methods.

We have created a reproducible and observable ERAS computational phenotype that reliably identifies ERAS cases that are actually ERAS cases and can be extracted from EMR data. This is a step forward in extracting the rich information in EMRs about how ERAS pathways are implemented, how well they are working, and how they can contribute to the improvement of surgical care.

## Additional File

The additional file for this article can be found as follows:

10.5334/egems.304.s1 Method verification cases. Classification of ERAS cases used in the verification step by each ERAS identification method examined.
